# Supplementary material for: Exosomes from Human Periodontal Ligament Stem Cells Promote Differentiation of Osteoblast-like Cells and Bone Healing in Rat Calvarial Bone
Source: Biomolecules. 2024 Nov 17;14(11):1455. doi: 10.3390/biom14111455 (PMC11591890; doi:10.3390/biom14111455)
Supplement: Supplementary file 1 [file biomolecules-14-01455-s001.zip › biomolecules-3193913-supplementary.pdf]

Exosomes from human periodontal ligament stem cells promote differentiation of osteoblast-like cells and bone healing in rat calvarial bone

Mhd Safwan Albougha, Hideki Sugii, Orie Adachi, Bara Mardini, Serina Soeno, Sayuri Hamano, Daigaku Hasegawa, Shinichiro Yoshida, Tomohiro Itoyama, Junko Obata and Hidefumi Maeda

Appendices

Materials and Methods

Checking the absence of mycoplasma contamination

The samples of cell culture were checked the absence of mycoplasma contamination using Takara PCR Mycoplasma Detection Set (Takara Bio Inc., Shiga, Japan). PCR was performed using Platinum™ *Taq* DNA Polymerase (Invitrogen, Waltham, MA, USA) in a PCR Thermal Cycler Dice (Takara Bio, Shiga, Japan). The control template in the kit was used as positive control.

Supplemental Figures

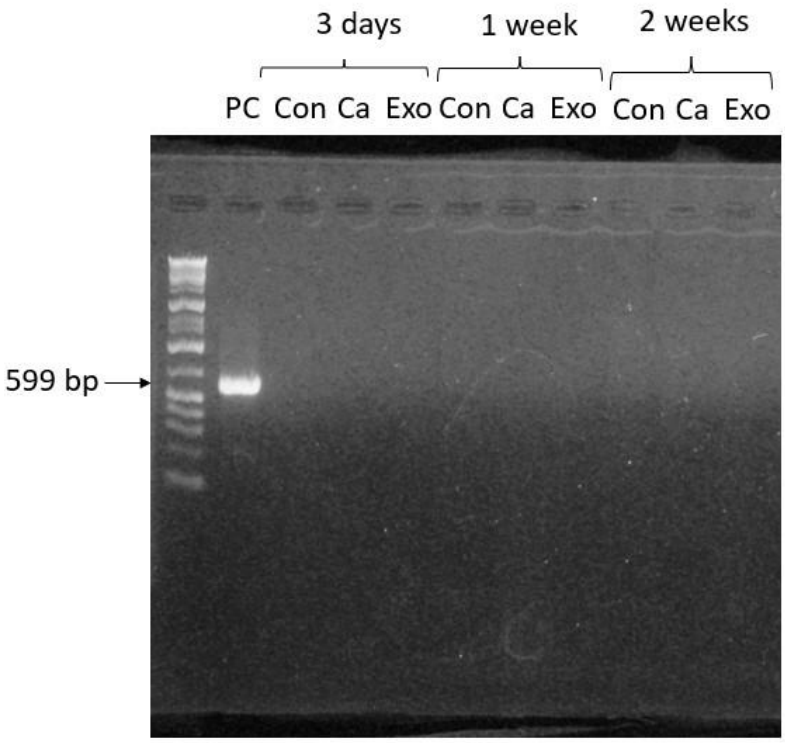

Supplemental Figure S1. Checking the absence of mycoplasma contamination

The samples from 3 days, 1 week and 2 weeks of culture were used for checking the absence of mycoplasma contamination. The following three conditions were selected ; α-MEM containing 10% Exosome depleted-FBS (Cont), Cont containing 1 mM $CaCl_2$ (Ca), Ca with 2 μg/ml PDLSCs-Exo (Exo). The control template in the kit was used as positive control (PC).

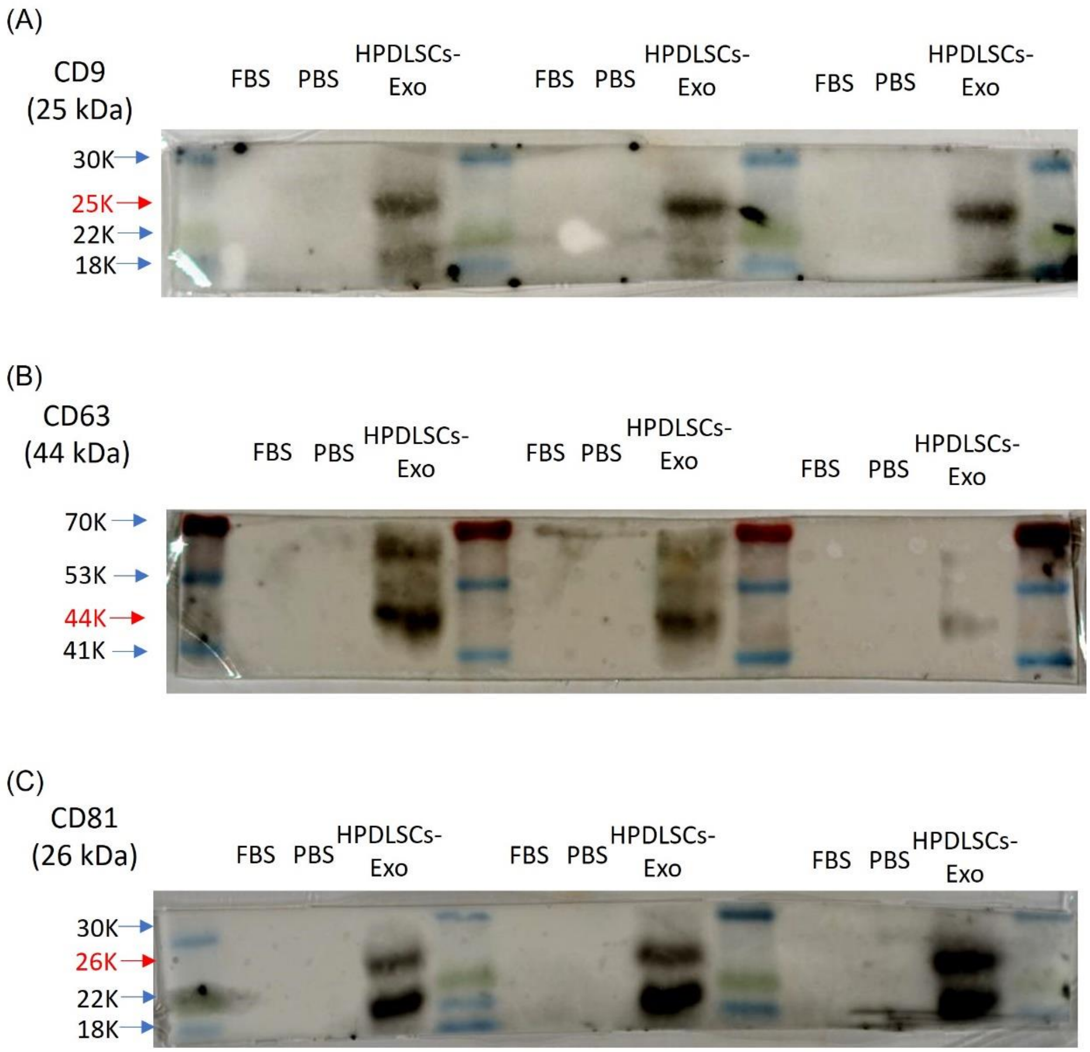

Supplemental Figure S2. Original images of western blotting analyses

(A-C) Original images of western blotting analyses in Fig.1C were shown. The blots of an anti-CD9 antibody (A; 25kDa), an anti-CD63 antibody (B; 44kDa) and an anti-CD81 antibody (C; 26kDa) were exhibited (n = 3).

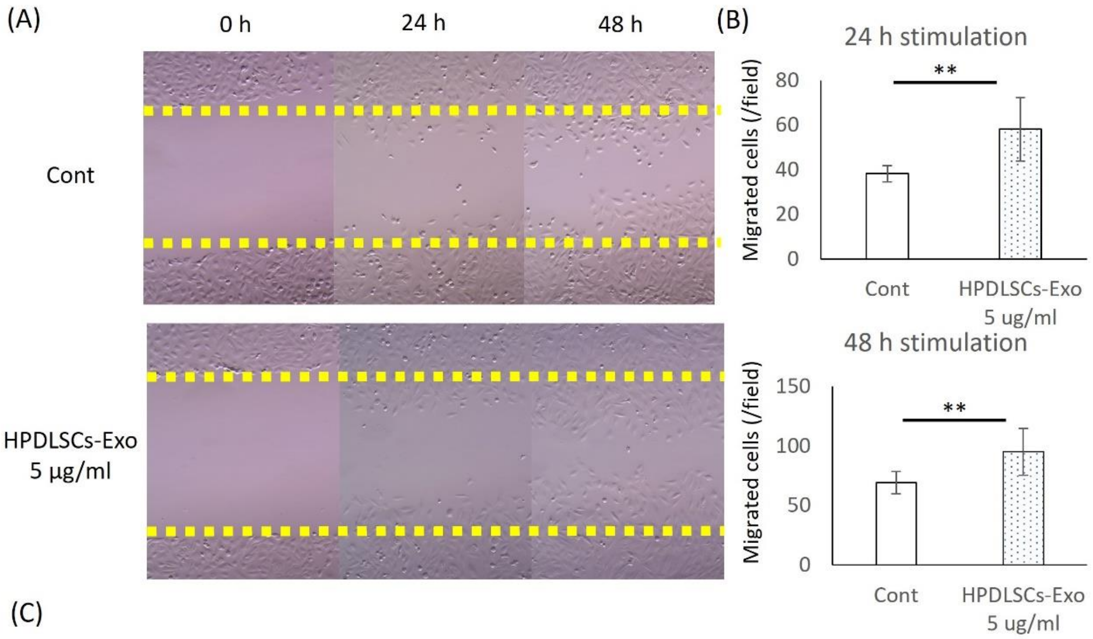

(C)

	Cont				HPDLSCs-Exo 2 ug/ml				HPDLSCs-Exo 5 ug/ml		
	0 h	24 h	48 h		0 h	24 h	48 h		0 h	24 h	48 h
Sample1	0	37	89	Sample1	0	80	121	Sample1	0	93	137
	0	38	79		0	44	89		0	73	108
	0	33	83		0	53	111		0	56	102
	0	38	72		0	62	117		0	62	97
Sample2	0	34	74	Sample2	0	77	88	Sample2	0	70	112
	0	34	63		0	74	98		0	54	116
	0	38	64		0	77	136		0	45	93
	0	44	69		0	52	89		0	61	105
Sample3	0	38	59	Sample3	0	44	81	Sample3	0	49	67
	0	43	58		0	46	74		0	66	100
	0	40	63		0	40	84		0	68	105
	0	41	81		0	34	72		0	58	72
Sample4	0	38	61	Sample4	0	64	119	Sample4	0	37	72
	0	44	65		0	64	99		0	48	89
	0	33	63		0	64	88		0	38	71
	0	38	66		0	52	84		0	52	76
Average	0	38.1875	69.3125	Average	0	57.9375	96.875	Average	0	58.125	95.125
standard deviation	0	3.581783	9.364605	standard deviation	0	14.41975	18.65431	standard deviation	0	14.2120840 6	19.59549268

Supplemental Figure S3 Effects of HPDLSCs-Exo on migration of Saos2 cells

(A) Saos2 cells were cultured in α-MEM containing 10% Exosome depleted-FBS (Cont) and Cont with 5 µg/mL of HPDLSCs-Exo. The scratch wound healing was observed at three time points after the scratch (0 h, 24 h and 48 h). n = 4. (B) Quantification of

migrated cells was performed. The values are shown as the averages of migrated cell numbers per well. n = 4, ***p* < 0.01. (C) The raw data of migrated cells. The values of four fields/well were exhibited.

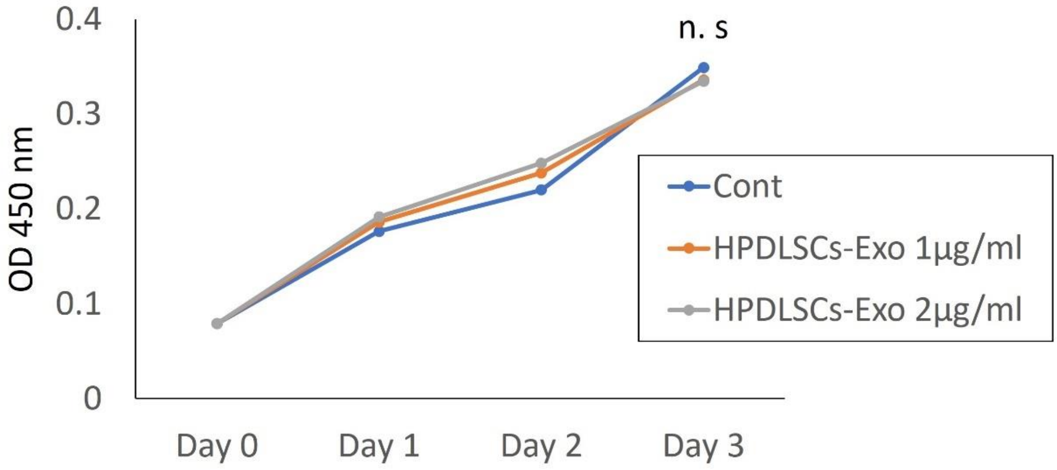

Supplemental Figure S4. Effect of HPDLSCs-Exo on proliferation of Saos2 cells

Saos2 cells were cultured in α-MEM containing 10% Exosome depleted-FBS (Cont) and Cont with 1 or 2 μg/ml HPDLSCs-Exo. WST1 cell proliferation assay was performed at 4 time points of treatment (day 0, 1, 2 and 3). Absorbance was measured at 450 nm. n=3. n. s = no significance.

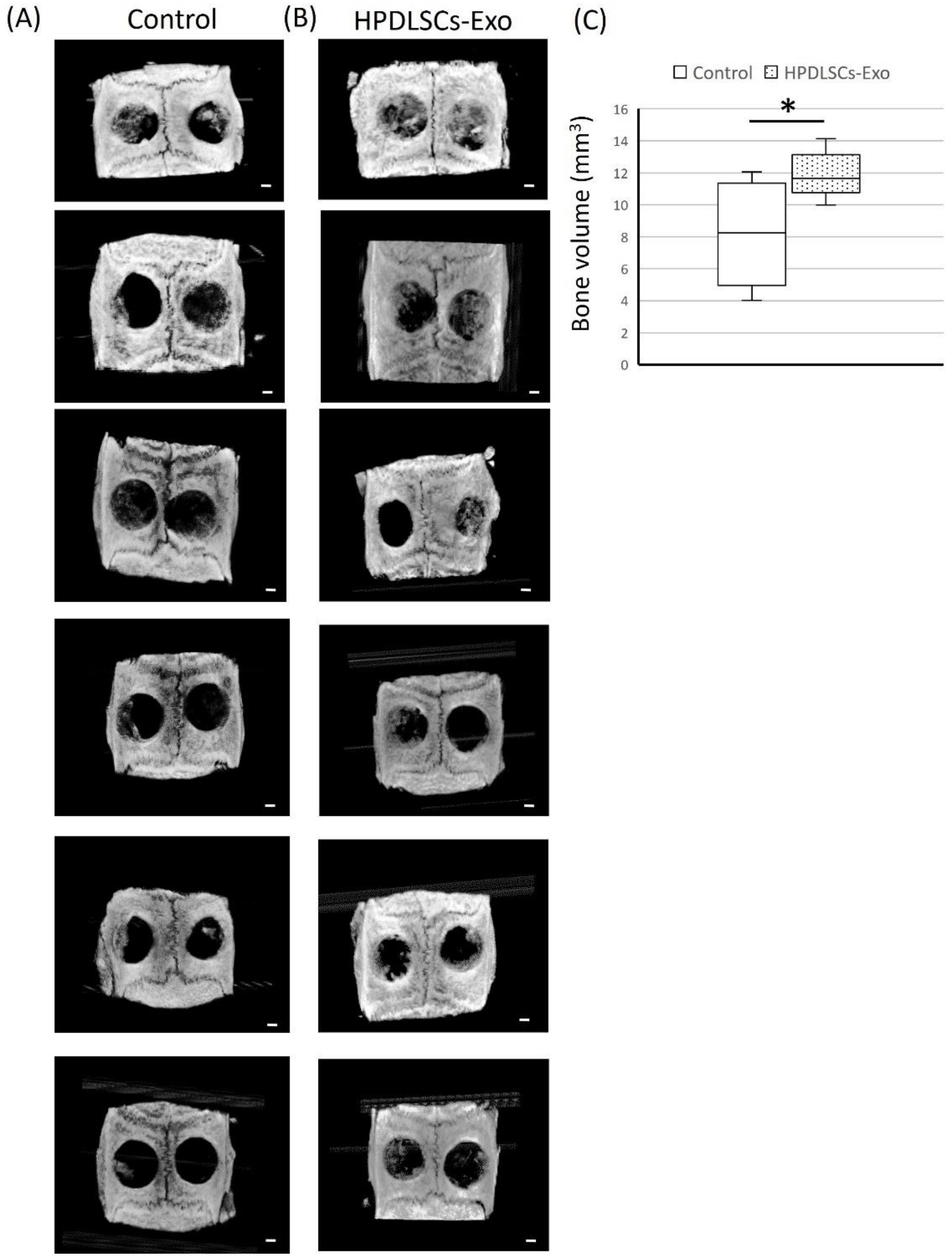

Supplemental Figure S5. Effects of HPDLSCs-Exo on bone healing in rat calvarial bone defect

(A, B) All micro-CT images of the defect site in rat calvarial bone treated with or without

HPDLSCs-Exo. The rats (12-weeks old, male) were anesthetized and two critical-sized calvarial defects (5 mm diameter) were created on each side of the calvarial bone using dental trephine bur. Hydrogel with PBS (Control) or 60 μg/ml PDLSCs-Exo loaded Hydrogel (PDLSCs-Exo) was injected into the defect site. Both defect sides were received same treatment. After 6 weeks of treatment, the samples were collected and the images of Control (A) and PDLSCs-Exo (B) were scanned by micro-CT. Bars = 1 mm. (C) The areas of newly formed bone in defect site were quantified. The outlier sample of HPDLSCs-Exo group was excluded. p*<0.05.

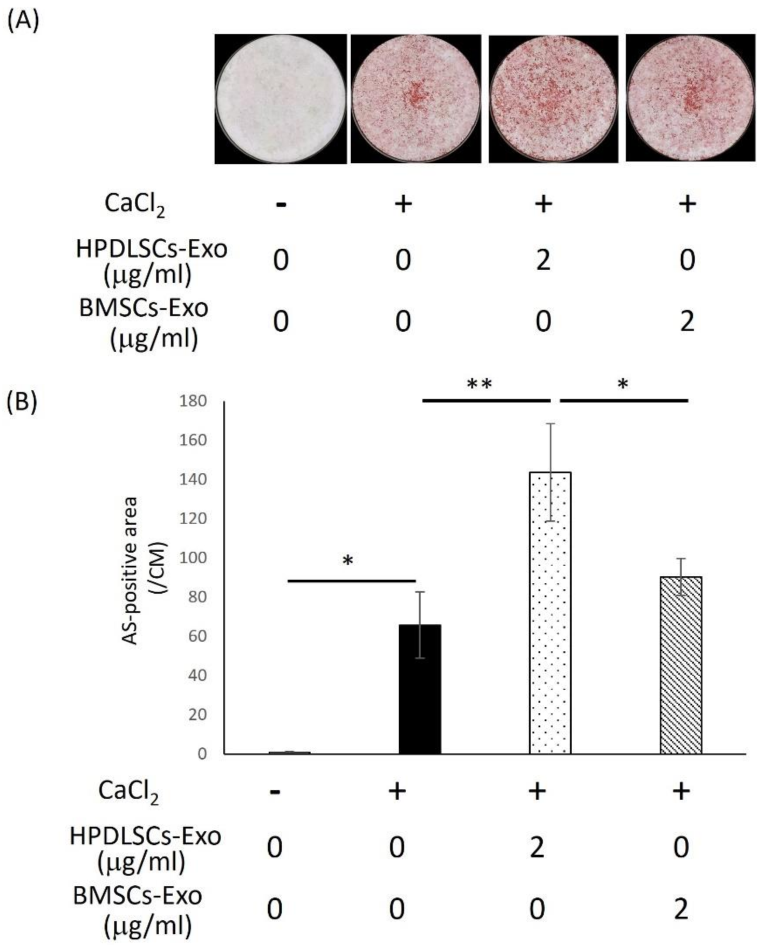

Supplemental Figure S6. Effect of HPDLSCs-Exo and BMSCs-Exo on on osteoblastic differentiation of Saos2 cells

(A) Saos2 cells were cultured in α-MEM containing 10% Exosome depleted-FBS (Cont), Cont containing 1 mM $CaCl_2$ (Ca), Ca with 2 µg/ml PDLSCs-Exo, and Ca with 2 µg/ml BMSCs-Exo. Alizarin red S staining (AS) was performed after 2 weeks of culture. (B) AS-positive areas were quantified. *p < 0.05, **p < 0.01, n=3.
